# Evaluating Somatic Experiencing^®^ to Heal Cancer Trauma: First Evidence with Breast Cancer Survivors

**DOI:** 10.3390/ijerph20146412

**Published:** 2023-07-20

**Authors:** Denise Vagnini, Massimo Maria Grassi, Emanuela Saita

**Affiliations:** 1 Department of Psychology, Università Cattolica del Sacro Cuore, 20123 Milan, Italy; 2 Breast Unit, Humanitas Gavazzeni Clinical Institute, 24125 Bergamo, Italy

**Keywords:** breast cancer, Somatic Experiencing, mind-body, group intervention, e-health, trauma, anxiety, depression, body image, coping strategies

## Abstract

Somatic Experiencing^®^ is a bio-psychological method for the treatment and prevention of trauma and chronic stress, which has never been investigated with breast cancer (BC) survivors. Eight weeks of web-based synchronous group sessions were structured between April and June 2022. Potential participants were recruited using a convenience sampling approach and through the collaboration of a public hospital in northern Italy and a non-profit association of BC women. Thirty-five eligible participants were enrolled and divided into an intervention group (n = 21) and a control group (n = 14). Anxiety, depression, distress (HADS), coping strategies (Mini-MAC), trauma reworking skills (PACT), and body image (BIS) were assessed at T0 and after 8 weeks (T1). Qualitative items concerning the most significant moments and learnings were completed at T1 by the intervention group. An independent *t*-test confirmed no between-group psychological differences at T0. As hypothesized, paired-sample *t*-tests showed decreases in anxiety, depression, distress (*p* < 0.05), and anxious preoccupation coping strategy (*p* < 0.001), but also improvements in forward focus (*p* < 0.05) and body image (*p* < 0.001) in the intervention group. The controls worsened over time with increases in hopeless/helplessness (*p* < 0.001) and avoidance (*p* < 0.05) coping strategies. Textual analyses extracted five dominant themes that summarized the meaning of the experience for participants. The preliminary results suggest the effectiveness of the intervention.

## 1. Introduction

Breast cancer (BC) is the most common cancer among women in western countries, and it is one of the types of cancer with a greater psychopathological impact, especially in terms of symptoms related to post-traumatic stress, which could remain for a long time, even after discharge from the hospital and medical treatments [1].

According to the scientific literature [2], the reception of the diagnosis and the memory of that experience are related to several symptoms, such as hyperarousal, emotional numbness, the feeling that it is happening to another person by themselves, intrusive thoughts, nightmares, and flashbacks. In addition, the severity of the diagnosis is positively correlated with increased risks of developing a fear of the future and of possible relapses, and, in some cases, avoidance coping strategies, deficiencies in information processing, problem-solving skills, and decision-making processes, or difficulties in communicating and expressing personal needs [3,4].

Moreover, women with BC can experience deep inner wounds, as the breast has strong symbolic meanings evoking, since ancient times, the concepts of femininity, motherhood, and sensuality that are deeply rooted in our culture. Often, patients receive a combined-type cancer treatment, and despite the improvements in surgical and treatment methods, it is undeniable that women’s bodies go through changes (e.g., hair loss, weakening and color change of nails), some of which are indelible (e.g., scars, alteration of the shape of the breast, or the need for breast implants) after illness. Consequently, these events could trigger a strong stress reaction and patients could be confronted with various psychological side effects, such as anxiety and depressive symptoms, compromised body image, and a perception of a lack of self-integrity. It is important to note that increased impairment of emotional tone and a decrease in overall quality of life could have negative effects on BC progression, survival rates, and adherence to treatments [5,6,7].

Life after illness is one of the most challenging and crucial aspects to take into account in follow-up settings. Dedicated attention to this aspect is needed, as life expectancy after BC has increased, and according to the Italian National Institute of Health (ISS, Istituto Superiore di Sanità) [8], today, the survival rate at 5 years from diagnosis is about 88%. However, rehabilitation is a very delicate stage, because it focuses on improving the woman’s psycho-physical well-being, regaining independence, and recovering full participation in life, work, and social contexts after illness.

As a result, additional resources must be allocated from this perspective to manage and support long-term BC survivorship. Further, it is important to identify effective interventions for the treatment of BC trauma that are aimed at responding to patients’ complex health-related issues [9,10,11,12,13].

For supportive care, BC survivors can benefit from complementary and integrative methods, and mind–body interventions. These practices, based on a bio-psycho-social approach to care, could have a decisive role in improving overall quality of life during survivorship, insofar as they associate sensitive, cognitive, motor, and affectional factors, and they intend to boost the brain’s capacity to positively affect bodily functions. This can happen through the involvement of bottom-up and top-down mechanisms, or a combination of both these processes [14,15,16,17]. In this way, mind–body practices could exert effects on the overall physiology of the body, such as reducing psychophysical symptoms, managing breathing difficulties, and reducing related fatigue [18].

Further, in some cases, these practices are proposed in group settings, including moments of discussion and comparison of lived experiences within a network of peers; a precious opportunity for learning functional skills for positive adaptation [19,20]. This setting helps group members to become aware of what is going on inside themselves when they “meet” others and share their reflections, because the presence of the individual is functional to the whole group, and vice versa. In this way, the group becomes a place safe enough to slow down, note “what is happening now”, and take the risk of experimenting with new ways of managing inner experiences and response modes [21].

Finally, with the advance of technology, research in the field of “e-health” and remote interventions has long been stressed, especially for group psychological or psycho-educational support programs for cancer patients, showing the first evidence of the potential of this modality [22,23,24]. However, further research is necessary to clarify the potential and benefits of these types of e-health interventions in the field of mind–body practices with BC survivors, as well as to pursue new treatments that can successfully complement the existing ones for patients with oncological diseases. Among these is the Somatic Experiencing^®^ Model.

### The Somatic Experiencing^®^ Model

Somatic Experiencing^®^ is a bio-psychological working model pioneered and developed by Peter Levine [25,26,27] for understanding, preventing, and treating post-traumatic stress disorder and chronic stress.

“Somatic” is a reference to the Greek word “soma”, referring to the living body, while “Experiencing” is a verb that alludes to the phenomenological level of being in the “here and now” [28].

From this theoretical perspective, Somatic Experiencing^®^ is a model of resilience that helps the subject develop intrinsic regulatory capacities and direct attention to visceral sensations (through interoception, i.e., literally sensing into the body) and musculo-skeletal feelings (through proprioception and kinesthesis) to increasingly tolerate and accept them (and related emotions) and activate resources to modify the stress response in an adaptive manner [29].

Basically, Somatic Experiencing^®^ is directed at the functioning of the core regulatory levels of the nervous system and in particular, it is directed at the two branches of the autonomic nervous system: the sympathetic one, dedicated to excitation, and the parasympathetic branch, responsible for relaxation [28]. Specifically, during a threatening event, the sympathetic nervous system is activated in a heightened state, and the parasympathetic nervous system also becomes activated to slow or shut down body systems. However, when this cycle hesitates in a state of unresolved immobility reaction, it can create a dissociation [14,28,29]. According to this, Somatic Experiencing^®^ was designed to be effective in cases of dissociation, dysregulations in the body, and neural networks that mediate the survival-oriented fight/flight/freeze responses, but also the reflective, symbolic, and integrative mental functions for the processing of the lived experience [30,31].

On these grounds, it prioritizes bottom-up processing (body–emotions–thought), starting from the body and the body memory linked to trauma to manage the hyperarousal [6]. The objectives are to work on the physical and psychological implications of trauma (e.g., distress and chronic fatigue) by helping the patient change the trauma-related stress response. According to the Somatic Experiencing^®^ model, trauma is not defined by any specific event in life, but it is considered an embodied response that reflects the subject’s inability to discharge survival energy (e.g., the subject remains in a freeze response as a state of immobilization or a feeling to be tapped) within a variety of situations in life. Somatic Experiencing^®^ practices are aimed at both providing safe physiological outlets for repressed energy and promoting psychological stability by correcting hyperarousal and ultimately stabilizing the autonomic nervous system. In this process, the individuals learn to connect with their own felt sense experiences, discover internal resources, and resolve traumatic tensions in a safe and supportive space [25,29,32].

Despite the high interest of clinical practice in this treatment, there is still little scientific production. Preliminary research on the Somatic Experiencing^®^ model studied its applications with different forms of trauma and in different contexts, e.g., post-traumatic stress disorders [33], natural disasters [34,35,36], chronic lower-back pain [37], chronic exposure to discrimination [38], and professionals who treat trauma [39]. However, up until now, it has not been presented in the oncological field. Specifically, there have been no studies that investigated the efficacy of the Somatic Experiencing^®^ model for BC survivors, neither in person nor online.

For these reasons, we designed a web-based Somatic Experiencing^®^ intervention in a group setting to study its effectiveness in the improvement of the psychological health of patients with previous BC, where psychological health is defined as the combination of psychological factors, coping skills, body image perception, and mental health disorders (i.e., anxiety and depressive symptoms), according to other research on health [40].

## 2. Aim and Hypothesis

The aim of this study was to evaluate changes in psychological health after a period of 8 weeks of web-based synchronous group sessions of Somatic Experiencing^®^ in the experimental group vs. the control group.

Starting from previous empirical evidence on Somatic Experiencing^®^ (see the Introduction), we hypothesized that more positive psychological health, trauma reworking and coping skills would be observed in the participants of the intervention group, while more difficulties in the processing of the oncological disease experience would be observed in the control group.

## 3. Materials and Methods

### 3.1. The Intervention

A modular structure in a group setting delivered on an online platform (i.e., Google Meet) was organized.

Enrolled participants of the intervention group (i.e., Somatic Experiencing^®^ sessions) were divided into three small groups. The small groups were functional to provide complete attention to the needs of all patients and allow them to be full protagonists of the program.

The intervention comprised 8 weeks of web-based synchronous group sessions of guided practices with a specialist that obtained certification following a triennial training as an operator of Somatic Experiencing^®^. The sessions took place once a week, lasted for about 60 min, and were all divided into two parts: (1) training on the acquisition of new skills; and (2) performing specific guided practices and sharing reflections within the small group. In addition, participants could also use an asynchronous group chat with a qualified psychologist, available both during sessions and during the time intervals between them.

At the end of each session, the participants also received a supply summary of the aims, the skills taught, and the practices performed in that specific class. This schedule was shared on the group chat and left available for download for patients, so that each participant could try again and improve the practices learned, even in everyday life.

The intervention, specifically dedicated to people at an entry level, was based on three crucial nodes of the Somatic Experiencing^®^ model: (1) defense strategies; (2) resources; and (3) primary techniques for the self-regulation of the organism. Table 1 describes the modular structure and the contents of each session.

### 3.2. Study Design and Participants

A pre-posttest design with two assessment times (T0: baseline; T1: post-intervention after 8 weeks) and two groups (an intervention and a control group on a waiting list) balanced for medical characteristics was implemented.

To be included, eligible participants had to be women, diagnosed for up to 5 years with mono- or bilateral BC (stage 0 to III without metastases or recurrence diagnosis at baseline), having undergone surgery (i.e., quadrantectomy or mastectomy), autonomous, with a school level of at least 5 years, be able to understand and express themselves in Italian, not engaged in a psychological or psychotherapeutic path at recruitment (and for the next 8 weeks of intervention), and who have never attended a Somatic Experiencing^®^ course (i.e., they had to be at an entry level). Patients with organic diseases related to the central nervous system (i.e., dementia or other neurodegenerative diseases), and with ongoing psychiatric disorders were excluded.

Using a convenience sampling approach, the research was proposed to potential participants identified in a public hospital and a non-profit association for BC survivors active in northern Italy (Lombardy region).

A priori power analysis for the *t*-test was performed using G*Power Software, version 3.1 [41,42], setting α = 0.05, power (1-β err prob) = 0.8, and allocation ratio N1/N2 = 0.5. A total sample of N = 42 (i.e., n = 28 subjects for the intervention group and n = 14 controls) was required to detect a medium effect size according to criteria [43].

**Figure 1:**
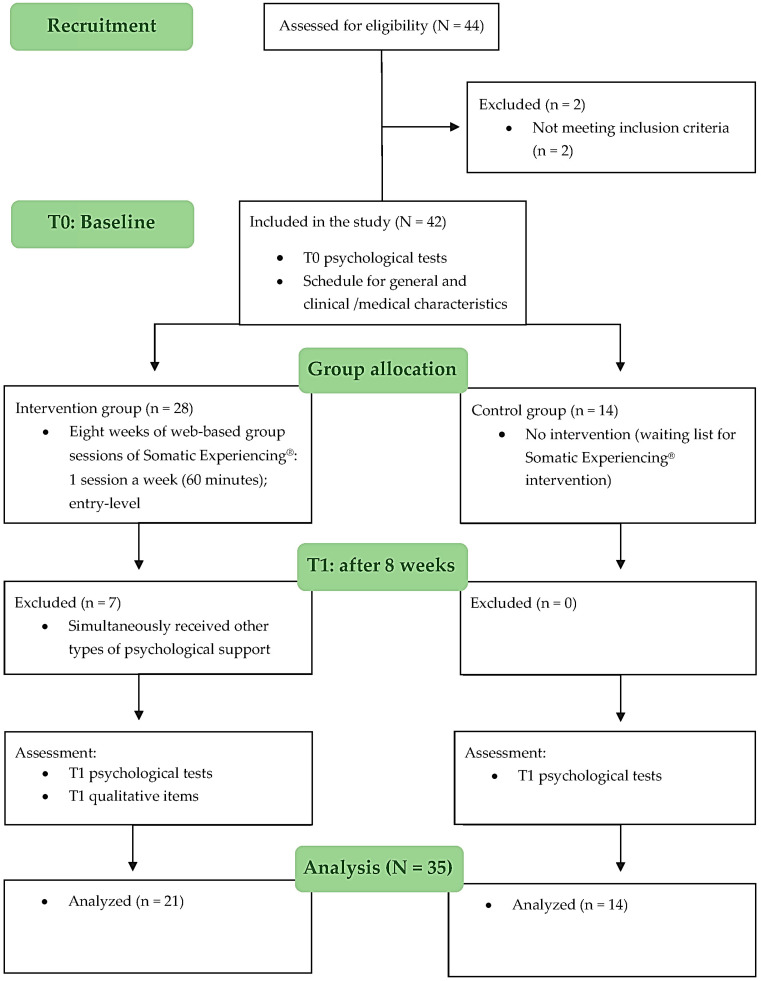
Flowchart.

Participants were recruited between December 2021 and April 2022 among those who had voluntarily chosen to participate. Forty-four women were assessed for eligibility and two were excluded, so forty-two women comprised the initial sample. Of these, n = 28 BC survivors completed the Somatic Experiencing^®^ intervention (8 weeks between April and June 2022), but n = 7 participants were removed from the analyzed sample because they simultaneously started another activity aimed at psychophysical support. Therefore, the total sample was composed of 35 participants: n = 21 comprised the intervention group, while the remaining n = 14 comprised the control group (women on the waiting list for Somatic Experiencing^®^ intervention). Figure 1 shows the research process.

This study was reviewed and approved on 4 November 2021 by the Ethics Commission of the Department of Psychology at Università Cattolica del Sacro Cuore, Milan, Italy (CERPS: Commissione Etica per la Ricerca in Psicologia), protocol No. 82-21.

### 3.3. Procedure and Data Collection

The survey for this research was implemented on the Qualtrics XM Platform™ (Qualtrics, Provo, UT), version from October 2021 (Copyright © 2022 Qualtrics), available at: https://www.qualtrics.com.

Data were collected through an online survey that took about 20 min and was distributed through an anonymous link. Before the beginning of the intervention (T0: baseline assessment) and after the end of the last session or after 8 weeks for the control group (T1: post-intervention assessment), a trained research collaborator using the private online group chats distributed to both the intervention and control subgroups the URL for the web-based survey. During both assessment phases, patients could ask research collaborators (using the group chat or a private chat) questions or technical problems with the survey.

Furthermore, a detailed Privacy Policy and a clear description of the aims and procedures of the study were included in the survey’s introduction, in accordance with the principles of the 1964 Helsinki Declaration and its later amendments. In addition, at the end of the same page, the e-mail contacts of the research Principal Investigator and collaborators were provided for specific further information. After reading and before starting the completion of the self-report questionnaires, the web survey site asked the participants to agree (i.e., give informed consent) to the terms and conditions of the research.

Participants did not provide names or contact details, and anonymity was guaranteed. The survey included a short algorithm for calculating a subject alphanumeric identification code (ID code), such that the online data were de-identified before data entry and the results were completely anonymous. Finally, the ID codes allowed us to blindly match the participants’ scores at T0 and T1.

### 3.4. Measures

At the baseline (T0) and after 8 weeks (T1), psychometric measures were collected to assess anxiety, depression, distress, individual coping strategies, trauma reworking skills, and perception of body image.

Only at T0, the survey also contained a schedule for general sample characteristics (i.e., age, marital status, dependent children, education level, and person recognized as the main caregiver) and clinical/medical information (i.e., year of diagnosis, type of surgery, and medical treatments).

Finally, only at T1, the intervention subgroup completed some short qualitative feedback on the efficacy of the proposed program. The participants were asked whether they deemed the approach of the intervention to improve well-being in BC survivors; specifically, they had to point out three significant moments of the intervention (item: “List the three most significant moments you have experienced”) and three important learnings from the intervention (item: “List the three most important learnings you have found out”).

Below, psychometric measures and Cronbach’s Alpha scores for the internal consistency of each scale in the current study are described, with accepted values of α ≥ 0.65 according to criteria for self-report questionnaires [44].

HADS: Hospital Anxiety and Depression Scale [45]. The Italian version of HADS [46] was used to assess anxiety, depression, and psychological distress related to stress and physical symptoms felt by patients during an organic disease, such as an oncological one. It is composed of a total score for distress (α = 0.905) and two seven-item Likert subscales with answers scored 0–3: HADS-A for anxiety (α = 0.890; example items: “Worrying thoughts go through my mind”; “I get sudden feelings of panic”) and HADS-D for depression (α = 0.806; example items: “I feel as if I am slowed down”; “I have lost interest in my appearance”). The total score of both subscales ranges from 0 to 21. The literature provides these cut-offs: a score of ≥11 defines the presence of psychological morbidity with an “abnormal” level of mood disturbances, scores of 8–10 are indicative of a “borderline” class, and scores of 0–7 characterize “normal” profiles.

Mini-MAC: the Mini-Mental Adjustment to Cancer scale [47]. The Italian version of the Mini-MAC Scale [48], consisting of 29 items scored on a 4-point Likert scale (from 1 = completely disagree to 4 = completely agree), was used to evaluate individual coping strategies adopted to cope with cancer disease. The subscales are: 1. Fighting Spirit (α = 0.729; example item: “I am determined to beat this disease”); 2. Hopeless/Helplessness (α = 0.902; example item: “I feel like giving up”); 3. Anxious Preoccupation (α = 0.875; example item: “I feel very angry about what has happened to me”); 4. Fatalism (α = 0.727; example item: “At the moment I take one day at a time”); and 5. Avoidance (α = 0.718; example item: “I distract myself when thoughts about my illness come into my head”).

PACT: Perceived Ability to Cope with Trauma [49]. The Italian version of PACT [50] consists of 20 items scored on a 7-point Likert scale (from 1 = not capable at all to 7 = extremely capable). It was used to measure trauma reworking skills through two subscales: Trauma Focus (α = 0.861; example items: “I reflect on the meaning of the event”; “Coping with the difficulties of life”) and Forward Focus (α = 0.928; example item: “Stay focused on my goals and plans”; “I remind myself that things will get better”). By combining the sum and the discrepancy score into a single variable, we obtained the flexibility score (range: 0–1), which indicates the patient’s ability to modify coping strategies according to requests of the environment and/or social context.

BIS: Body Image Scale [51]. The Italian version of BIS [52] was used to measure perceived body image among patients after surgery (α = 0.912). It consists of 10 items on a 4-point Likert scale (from 0 = not at all to 3 = very much), and example items are: “Have you felt less physically attractive as a result of your disease or treatment?”; “Have you been feeling the treatment has left your body less whole?”. The final score range is 0–30 and higher scores correspond to more negative concerns about body image. As the literature does not provide intermediate cut-offs for the interpretation of clinical aspects, total scores were organized into three categories according to previous studies conducted by authors [11,53]. In particular, we classified: “good body image” (scores 0–10), “composite body image” (scores 11–20), and “impaired body image” (scores 21–30).

## 4. Data Analysis

Psychometric data were analyzed using IBM SPSS, version 27.0 [54]. Firstly, we performed data screening by checking missing responses for each item. Subsequently, descriptive statistics (i.e., frequency, mean, and SD), chi-squared tests, and independent *t*-tests (α = 0.05 two-tailed) were used to describe the sample characteristics and between-group differences at baseline (T0).

Normality of distributions was assessed using the Shapiro–Wilk test, setting α = 0.05 as a threshold value.

Paired *t*-tests (setting α = 0.05) were used to study the mean differences pre–post-intervention (T0-T1) within each group and to verify our hypothesis (i.e., to observe better psychological conditions, trauma reworking and coping skills in the intervention group vs. controls). Cohen’s d effect sizes were provided for all findings according to the following criteria: d = 0.2 small effect; d = 0.5 medium effect; d = 0.8 large effect [43,55].

Finally, we analyzed qualitative items on the three most significant moments and the three most important learnings according to the participants. We chose an inductive approach [56], in which all emerging themes resulted from the words of the participants, rather than from preconceived theoretical concepts. Two authors (D.V. and E.S.) independently performed data analysis using the paper-and-pencil method, and possible inconsistent coding decisions were discussed by the coders to reach consensus (Cohen’s k ≥ 0.80) [57]. Meaningful verbatim quotes were translated into English by the authors and presented to support the extracted dominant themes.

## 5. Results

All participants completed the survey and there were no drop-outs or missing responses.

### 5.1. Sample Characteristics

The participants were N = 35 women of Italian nationality, all diagnosed with monolateral BC (stage I to III) and resident in the north of Italy.

The independent *t*-tests and chi-squared tests showed no baseline differences in medical features (i.e., year of diagnosis, type of surgery, and type of medical treatments), clinical characteristics (i.e., body image, anxious, and depressive levels), or general characteristics (i.e., age, presence of children, educational level, and figure considered the main caregiver), except for marital status (χ^2^_(2)_ = 14.620, *p* < 0.001, Cramer’s V = 0.646).

The sample characteristics by group and baseline comparisons are presented in Table 2.

### 5.2. Group Comparisons

The independent *t*-test on psychometric variables (i.e., anxiety, depression, distress, fighting spirit, hopeless/helplessness, anxious preoccupation, fatalism, avoidance, forward focus, trauma focus, flexibility, and body image) showed no significant (*p* < 0.05) baseline (T0) differences between the intervention group (n = 21) and control group (n = 14). Table 3 summarizes the findings.

Considering the Somatic Experiencing^®^ intervention group, the paired *t*-tests showed a significant (*p* < 0.05) decrease in anxious (t_(20)_ = 2.788, *p* = 0.011, Cohen’s d = 0.608), depressive (t_(20)_ = 2.682, *p* = 0.014, Cohen’s d = 0.585), and distress (t_(20)_ = 3.107, *p* = 0.006, Cohen’s d = 0.678) symptoms.

Taking into account the cut-off criteria provided by the literature for the interpretation of HADS (Hospital Anxiety and Depression Scale) scores [45,46], at T1, n = 17 (80.95%) participants had “normal” levels of anxiety, n = 3 (14.29%) participants were at the “borderline” level, and only one woman (4.76%) had “abnormal” symptoms. The majority of participants (n = 19; 90.48%) had “normal” levels of depressive symptoms, n = 2 (9.52%) participants were at the “borderline” cut-off level, and no participants were classified in the “abnormal” symptoms’ class.

They also reported a significant (*p* < 0.001) decline in recourse to anxious preoccupation as a coping strategy (t_(20)_ = 4.198, *p* = 0.0001, Cohen’s d = 0.916) and an improvement (*p* < 0.05) in forward focus (t_(20)_ = −2.920, *p* = 0.008, Cohen’s d = −0.637) as a trauma reworking skill. Finally, they showed a significant enhancement (*p* < 0.001) in body image perception (t_(20)_ = 6.234, *p* = 0.0001, Cohen’s d = 1.360) compared with baseline (T0).

Considering the BIS (Body Image Scale) [51,52] ad hoc cut-off criteria [11,53], at T1 most of the participants (n = 18; 85.71%) showed a “good body image”, n = 3 (14.29%) participants reported a “composite body image”, and nobody perceived an “impaired body image”.

The results confirm our hypothesis, according to which we expected to observe more positive psychological health conditions, trauma-reworking and coping skills in the participants of the intervention group. Furthermore, the psychological health condition of the controls did not remain stable compared with the baseline (T0) assessment.

In fact, the participants of the control group worsened over time, showing a significant (*p* < 0.001) increase in hopeless/helplessness (t_(13)_ = −5.192, *p* = 0.0001, Cohen’s d = −1.388) as a coping strategy and a significant (*p* < 0.05) increase in the avoidance of disease or its consequences (t_(13)_ = −3.397, *p* = 0.005, Cohen’s d = −0.908).

Comparisons within groups are summarized in Table 4.

### 5.3. Qualitative Findings at the End of the Intervention (n = 21, Somatic Experiencing^®^ Group)

All 21 participants of the intervention group described three significant moments and three important learnings each, for a total of 126 responses (n = 63 significant moments and n = 63 important learnings, respectively).

Textual analysis of the three most significant moments of the experience led to the identification of three dominant themes, specifically dedicated to a new way of connecting with oneself and understanding one’s emotions related to the experience of illness but also to medical treatments, the awareness that the Somatic Experiencing^®^ sessions were useful to improve well-being and psychological state, and the group experience.

Finally, important learnings reported by participants were summarized in two main themes concerning two personal warnings for future life. Table 5 synthetizes these findings.

## 6. Discussion

This is a preliminary study aimed at investigating, for the first time, the efficacy of the Somatic Experiencing^®^ model and its entry-level practices in improving the psychological health, trauma reworking and coping skills of Italian BC survivors.

As the recent scientific literature indicates [14,15,16,17], research on trauma should not consider the body or the mind alone, but the two together as inseparable parts of the whole complexity of an individual. This approach holds together the neurobiological basis of stress, the subjective perception and response to threatening life events, and related psychological symptoms. Mind–body interventions are extremely relevant in the management of patients during the survival phase to decrease psycho-physical symptoms related to post-traumatic arousal and restore healthy functioning in daily life [18].

Based on our preliminary results, Somatic Experiencing^®^ could successfully complement the core of these existing interventions already proposed for cancer survivors.

Our sample consisted of BC survivors who, at baseline, had a composite to impaired body image and normal to borderline levels of anxiety and depression. In recent studies [5,58,59], this symptomatic framework has been a topical issue and it often confers worse outcomes in terms of prognosis and a higher mortality rate in those who deserve adequate clinical attention and targeted psychological support interventions. The women in our intervention group showed a better psychological condition at the end of the eight online group sessions. First, their levels of anxious, depressive, and distress symptoms significantly decreased. Similarly, a previous longitudinal investigation of a Somatic Experiencing^®^ intervention with professionals who treat trauma (i.e., health care providers) [39] reported a significant reduction in anxiety and somatization symptoms, and a significant improvement in physical and intrapersonal well-being. Also, a pretest–posttest within-group study [38] with gender non-conforming individuals chronically exposed to discrimination and social injustice highlighted a meaningful impact of ten sessions of Somatic Experiencing^®^ in a psychoeducational group setting on depressive and somatic symptoms, and global health quality of life. Further, a 15-session randomized study with a control group on a waiting list designed to reduce post-traumatic stress disorder [33] highlighted a reduction in depressive and post-traumatic distress symptoms in the intervention group vs. controls. It is important to underline that the protocols and the duration of the scheduled sessions in these researches were different. However, in line with preliminary studies, this first evidence highlights the positive and encouraging effects of our intervention.

Second, the results suggest that our modular intervention could promote the use of more functional coping strategies and trauma reworking skills in terms of a reduction in anxious preoccupation and higher forward focus, operationalized as facets of resiliency, adaptive competencies, and flexibility. Resiliency indicators also decreased in a study [35] that applied a brief version (about two) of Somatic Experiencing^®^ sessions with social service workers who were survivors of Hurricanes Katrina and Rita. Following the same application in the context of natural disasters, another study [36] researched Somatic Experiencing^®^ (75 min of practices) with one hundred and fifty tsunami victims in southern India, and they observed an improvement in well-being in terms of less intrusive and hyperarousal symptomatology and a reduction in the avoidance coping strategy.

Third, we observed that the proposed work with and through the body assisted women with BC in regaining their sense of security to live in their own bodies and removing the threat that comes from the body (i.e., represented by BC). In fact, they showed a decisive improvement in their body image, and the majority (about eighteen participants to twenty-one) were satisfied with their own body image. No other scientific evidence on the effectiveness of Somatic Experiencing^®^ practices studied the effect on this construct, and further research is necessary. However, according to the literature, body image is defined as a complex mental construction that combines one’s perception of the whole body and its parts, its movement and limits, subjective thoughts and evaluations, and the deeper feelings and emotions experienced regarding it [5]. Therefore, it is reasonable that the proposed intervention also deeply impacted this dimension, strictly correlated with emotional tone and quality of relational life, as evidenced by previous research with patients and dyads [4,11].

Considering the participants in the control group, they significantly worsened in the use of coping strategies, showing greater hopeless/helplessness and avoidance tendencies. The hopeless/helplessness coping strategy is among the most frequently found maladaptive conditions in cancer patients, associated with a greater presence of psychological distress, lower general quality of life, and dysfunctional adaptation to the disease condition in the long term. In short, it is related to a potentially compromising sequelae of several psychological and physical implications that significantly affect the living conditions of cancer patients [60]. Similarly, high levels of avoidance and intrusion tend to cause difficulties in the healing process [2]. Although these are only preliminary results, these outcomes appear alarming for the psychological condition of BC patients without a standard treatment for psychological support during the survivorship phase. These data stimulate us as a community of researchers, but also of clinical psychologists and psycho-oncologists, responsible for proposing easily and promptly applicable solutions for the long-term psychological support of this population.

Furthermore, we aimed to identify method-specific key factors of the proposed intervention to identify the strengths (vs. limits) and outline avenues for future research. In light of the positive qualitative feedback obtained from the intervention group, we could observe a generally positive evaluation and high satisfaction with the intervention and its outcomes. The dominant themes showed the perception that the intervention worked, a positive connection with body sensations and emotions, and the ability to live in the present moment and take care of oneself as the key elements that participants took home. In addition, they also recognized the reflection within the group as a meaningful moment of the program. This suggests a positive supportive function of the group, which became a space for participants (as peers) to discuss and share their experiences. Therefore, the group setting appeared as a valid alternative through which the intervention could be provided. This is in line with previous research [61,62,63,64] that assessed the effectiveness of the group setting and the modular intervention’s modality, with different populations including adult cancer survivors, highlighting high levels of participants’ satisfaction, quality of life, well-being, perceived confidence, and lower loneliness.

Regarding the possibility to use the online mode, in the last few years and even more during the pandemic period, the interventions of e-health in psycho-oncological care and the opportunities they offer have greatly increased [65]. Research has highlighted positive results of e-health programs, both in terms of information support (i.e., exchange of information between peers) and of strengthened coping, self-management, and empowerment skills [66]. In addition, according to the literature [67], the online mode could be considered a beneficial addition, if not even a cost-effective alternative.

To conclude, special attention is given to the fact that, in our sample, there were no drop-outs, but seven participants who had previously enrolled for the intervention were excluded because of their simultaneous adherence to other support interventions. These data suggest, on the one hand, the effectiveness of the intervention as described in this study (i.e., group and online settings, a modular structure of 8 weeks, the combination of a training and a performing part, and also the possibility for controls to be part of the intervention at the end) to involve participants and keep them engaged throughout the process. On the other hand, we cannot fail to note a need for extended, generalized, and immediate psychological support for the seven women who were excluded. As mentioned above, this should be taken into account in a more general reflection on the tools available to patients to be active agents in taking charge of their mental health during survivorship and how health professionals direct them in choosing the most suitable support path.

### Limitations and Future Directions of Research

The results must be interpreted with caution because of the convenience sampling method, the small sample size, the no-randomization design, the use of only self-report measures, and the absence of follow-up assessments that affect this initial investigation.

Then, according to the literature [68], we must consider that women who choose to participate in intervention programs have peculiar characteristics (e.g., intrinsic motivations and autonomy), which are different from those of BC survivors who do not choose to be engaged. For this reason, it would be appropriate to investigate how these attitudes affected the outcomes of the intervention.

Finally, as shown, the scientific panorama on Somatic Experiencing^®^ does not intercept a single target, but there is a heterogeneous compound that testifies to the pluri-adaptability of this technique in contexts where there is a potentially traumatic condition or a dysregulation of the autonomic nervous system. However, previous studies applied this intervention by adopting diversified protocols in terms of different number and durations of the scheduled sessions.

For these reasons, more research into Somatic Experiencing^®^ application with BC survivors and its methods and mechanisms is needed. Future controlled trials should overcome the limitations of the present study by evaluating short- and long-term prospects and their association with possible biological markers and physiopathological mechanisms [69] to corroborate the efficacy of this intervention.

## 7. Conclusions

The conceptualization of cancer, specifically BC, as a potentially traumatic event of great impact on people’s lives has opened up the possibility of using the Somatic Experiencing^®^ model in an area not explored until now. In fact, this is the first study that investigated the effectiveness of the Somatic Experiencing^®^ model on the improvement of psychological health, trauma reworking and coping skills of BC survivors. As shown, at the end of the intervention, the participants decreased their anxious, depressive, and distress symptoms, and their use of anxious preoccupation as a coping strategy. Further, they improved their orientation to the future (i.e., forward focus), and their body image perception. In contrast, the control group worsened, highlighting poor health conditions, such as a greater use of hopeless/helplessness and avoidance coping strategies.

Further, the strengths of our current research contributed, for the first time, to deepening the potential and benefits of Somatic Experiencing^®^ practices proposed in group settings and as an e-health modality during the BC survivorship phase.

To conclude, these preliminary results are encouraging. Somatic Experiencing^®^, as a treatment that could be effective in low dosages (and be less costly), could become a corollary technique for the treatment of the psychological implications that accompany BC medical care and long-term treatments.

## Figures and Tables

**Table 1:** Somatic Experiencing^®^ in online groups: Modular structure of the entry-level intervention for BC survivors.

Module	Aims	Practices and Skills
[Welcome] (1) Breathing	Developing awareness that changes in breathing are normal when we perceive an external threat. Learning strategies to calm down and send an important message to the nervous system: there is “No Danger”. Learning that the way we breathe is what makes the difference.	Conscious breathing to achieve relaxation responses in the body: inhale and exhale following the practitioner’s guidance. The ultimate goal of any breath work practice is to activate the peripheral nervous system.
(2) Grounding	Grounding practices help to connect to the present. They also allow the body to find its inner balance and to be physically and emotionally in contact with what is really happening in the “here and now” in order to:	Finding balance on your own legs, noticing how your feet make contact with the ground. Various practices can be explored, for example:
	- Feel safe in the body and trust the body’s inner wisdom; - Understand the overwhelming feelings caused by events from the past or any fear or emotions related to the future; - Learning that trauma is not in the event, but in the residual energy in the nervous system: regaining a sense of safety and discharging the energy in the body is crucial for self-regulating.	- Sitting position: pushing your hands on your knees; - Standing position: keeping your knees slightly bent with your hand pushing on them, or gently letting your body move left and right. Imagine a strong, steady tree, and stepping into this tree, where your arms become the branches, your body is the trunk of the tree, and your legs and feet become the root system: hands and arms can be along the sides of your legs and you can feel your feet on the floor.
(3) Felt-Sense	Felt-sense describes internal bodily awareness of the changes that our internal landscape goes through (i.e., senses, emotions, and feelings). It stimulates a healthy mind–body connection and helps the body to feel safe from within. Awareness is key to:	- Orienting: orienting yourself in the room and, therefore, the present and finding that it is a safe place to be; - Grounding: feeling the body weight on the chair, the back supported by it, and the legs, and make contact with your feet touching the floor; - Skin contact: touch, squeeze, pinch, and rub the skin on your hands, arms, and legs, and rub your hands together; - Movement: gently move and twirl your wrists, elbows, shoulders, and ankles, and stretch your knees to perceive where the body is in your own space; - “Butterfly Hug”: Cross your arms over your chest like you are hugging yourself. With your hands open, tap alternatively 25 times. Then, take a deep breath and see how you feel. You can repeat the tapping 2/3 times if needed.
	- Reconnecting to your body sensations and the inner gut instinct and intuition without any conscious thinking or judgement; - Learning to understand the body’s signals (e.g., an unusual feeling in the stomach or in the throat). They are physical sensations that arise in relation to an event or other circumstances (you can ask yourselves: “What do I feel?”).	
(4) Movement	Learning that the body has a memory and how emotions and sensations find their way to expression. Reconnect the body to movement as the antidote to the freeze response and, therefore, immobility, and provide flexibility back to the body to relieve tension and pain. Provide the body a new experience as it can move and protect itself, and reduce, relieve, or eliminate the debilitating symptoms of trauma stuck in the body (i.e., “frozen”).	Gentle movement practices, and self-touch where the stress is stuck in the body and release the tension and pain:
		- If the pain/tension is in the chest and throat, movement can help through your arms; - If the pain/tension is in the belly, movement can help through your legs; - If the pain/tension is in the head, movement can help through your jaws, mouth, and neck.
(5) Healthy Boundaries	Gain self-awareness and establish healthy boundaries, crucial for self-care and positive relationships. Strengthen your boundary awareness by exploring the cues in your body that give you feedback about your needs or limits. Setting clear, but flexible, boundaries involves listening to and respecting your somatic cues. Strong sensations often serve as important signals that can help you to recognize what is right or wrong for you and allow you to gain a personal space where you can be confident and protect yourself (e.g., learn to say “No” when necessary).	Protect your space: Place your right hand on your left shoulder and vice versa, and feel your space. Push hands against the wall: Regain confidence about your safe space and body, and the capacity to keep the required distance in various circumstances.
(6) Self-regulation	Learn how the body is able to self-regulate and how the autonomic nervous system receives information about the body and external environment, and how it responds by stimulating body processes. Detect tension and pain as a result of negative emotions or thoughts, and relieve the stress response through self-regulation resources and techniques.	Self-regulation is when you learn and develop your own tools to calm yourself down. Self-regulation tools are taught to help you move through big emotions and feelings, and find stabilization. By recognizing the body’s signals, you can facilitate energy discharge under the practitioner’s guidance. Tune into your body through:
		- Conscious breathing; - Tapping, squeezing, self-touch; - Regulating the body before talking about what causes tension to avoid enhancing the sensation and activating it.
(7) Freeze Response	Understanding that unsolved trauma symptoms develop from the freeze response, when trapped energy does not get discharged, but is stuck in the body: e.g., dissociation or immobility (paralysis, denial), debilitating emotional states (e.g., panic attacks, hypervigilance, and impotence), and chronic fatigue. Understanding medical trauma and the best way to avoid it, especially in conditions of recurrence of the disease and during medical procedures. Learning how to heal medical trauma by adopting the right techniques and resources.	Understand what happens in the body when you experience fear or anxiety (e.g., during medical procedures), and self-regulate or prepare to reduce or avoid trauma symptoms. Understand the dilemma of rationally wanting to obtain treatment and the survival instinct that is trying to escape it. Passive acceptance to avoid medical trauma is key.
(8) Recall Session: Questions, Comments, and Conclusion [Greetings]	Discussion of key topics and allowing everybody to comment and share. Discussing changes and quality of life awareness.	Get in touch with your own body and the available resources. Share your personal inventory of resources and recognize who you can ask for help. Breathing practice.

**Table 2:** Sample characteristics.

	Intervention Group (n = 21)	Control Group (n = 14)	Baseline (T0) Differences
Age—M (SD) range	54.14 (5.51) 39–62	50.00 (8.99) 33–66	t_(33)_ = 1.695, *p* = 0.100, Cohen’s d ^a^ = 0.585
Marital status—n (%) Single Married Cohabitation	5 (23.81) 15 (71.43) 1 (4.76)	1 (7.14) 4 (28.57) 9 (64.29)	χ^2^_(2)_ = 14.620, *p* = 0.0001 **, Cramer’s V ^b^ = 0.646
Children—n (%) Yes No	12 (57.14) 9 (42.86)	9 (64.29) 5 (35.71)	χ^2^_(1)_ = 0.179, *p* = 0.673, Cramer’s V ^b^ = 0.071
Educational level—n (%) Elementary school Middle school High school University	0 (0) 5 (23.81) 10 (47.62) 6 (28.57)	0 (0) 3 (21.43) 7 (50.00) 4 (28.57)	χ^2^_(2)_ = 0.031, *p* = 0.985, Cramer’s V ^b^ = 0.030
Diagnosis: Year—n (%) 2016–2017 2019–2020	10 (47.62) 11 (52.38)	3 (21.43) 11 (78.57)	χ^2^_(1)_ = 2.468, *p* = 0.116, Cramer’s V ^b^ = 0.266
Surgery: Type—n (%) Quadrantectomy Mastectomy	15 (71.43) 6 (28.57)	11 (78.57) 3 (21.43)	χ^2^_(1)_ = 0.224, *p* = 0.636, Cramer’s V ^b^ = 0.080
Medical treatments—n (%) Radiotherapy Chemotherapy Hormonal therapy Radio. and hormonal therapy Chemo and hormonal therapy Radio, chemo, and hormonal therapy No treatments	0 (0) 0 (0) 3 (14.29) 11 (52.38) 2 (9.52) 4 (19.05) 1 (4.76)	1 (7.14) 1 (7.14) 0 6 (42.86) 3 (21.43) 3 (21.43) 0	χ^2^_(6)_ = 6.681, *p* = 0.351, Cramer’s V ^b^ = 0.437
Main caregiver—n (%) Father/mother Other family member A friend Romantic partner/husband	1 (4.76) 3 (14.29) 3 (14.29) 14 (66.66)	1 (7.14) 1 (7.14) 1 (7.14) 11 (78.58)	χ^2^_(3)_ = 1.000, *p* = 0.801, Cramer’s V ^b^ = 0.169
Body image (T0: BIS)—n (%) Good body image (scores 0–10) Composite body image (scores 11–20) Impaired body image (scores 21–30)	6 (28.57) 11 (52.38) 4 (19.05)	1 (7.14) 6 (42.86) 7 (50.00)	χ^2^_(2)_ = 4.646, *p* = 0.098, Cramer’s V ^b^ = 0.364
Symptoms of anxiety (T0: HADS-A)—n (%) Normal level (scores 0–7) Borderline level (scores 8–10) Abnormal level (scores 11–21)	12 (57.14) 3 (14.29) 6 (28.57)	3 (21.43) 6 (42.86) 5 (35.71)	χ^2^_(2)_ = 5.303, *p* = 0.071, Cramer’s V ^b^ = 0.389
Symptoms of depression (T0: HADS-D)—n (%) Normal level (scores 0–7) Borderline level (scores 8–10) Abnormal level (scores 11–21)	16 (76.19) 5 (23.81) 0 (0)	8 (57.14) 6 (42.86) 0 (0)	χ^2^_(1)_ = 1.414, *p* = 0.234, Cramer’s V ^b^ = 0.201

*Abbreviations*: BIS, Body Image Scale; HADS-A, Hospital Anxiety and Depression Scale—Anxiety subscale; HADS-D, Hospital Anxiety and Depression Scale—Depression subscale. ^a^ Cohen’s d: d = 0.2 small effect; d = 0.5 medium effect; d = 0.8 large effect [43]. ^b^ Cramer’s V: 0.1 = small effect; 0.1 < V ≤ 0.3 = medium effect; 0.3 < V ≤ 0.5 = large effect [43]. ** *p* < 0.001.

**Table 3:** Baseline (T0) comparisons between groups: Independent *t*-test.

	Intervention Group (n = 21) T0: Baseline	Control Group (n = 14) T0: Baseline	Independent *t*-test		ES ^a^
Measures (Range)	M (SD)	M (SD)	t_(df)_	*p* Value	Cohen’s d
Anxiety: HADS-A (0–21)	7.57 (4.06)	9.14 (2.68)	−1.272_(33)_	0.212	−0.439
Depression: HADS-D (0–21)	4.76 (3.08)	6.64 (2.31)	−1.946_(33)_	0.060	−0.671
Distress: HADS (0–42)	12.33 (5.56)	15.79 (4.17)	−1.743_(33)_	0.091	−0.602
Fighting spirit: Mini-MAC (1–4)	3.06 (0.58)	2.95 (0.72)	0.515_(33)_	0.610	0.178
Hopeless/helplessness: Mini-MAC (1–4)	1.54 (0.52)	1.91 (0.68)	−1.805_(33)_	0.080	−0.623
Anxious preoccupation: Mini-MAC (1–4)	2.07 (0.67)	2.47 (0.52)	−1.893_(33)_	0.067	−0.653
Fatalism: Mini-MAC (1–4)	3.04 (0.60)	2.92 (0.67)	0.539_(33)_	0.593	0.186
Avoidance: Mini-MAC (1–4)	2.90 (0.82)	2.46 (0.80)	1.571_(33)_	0.126	0.542
Forward Focus: PACT (1–7)	5.19 (1.12)	4.50 (1.29)	1.702_(33)_	0.098	0.587
Trauma Focus: PACT (1–7)	4.91 (1.22)	4.87 (1.22)	1.109_(33)_	0.914	0.038
Flexibility: PACT (0–1)	0.86 (0.06)	0.82 (0.08)	1.701_(33)_	0.098	0.587
Body image: BIS (0–30)	14.43 (6.98)	18.64 (5.08)	−1.940_(33)_	0.061	−0.669

*Abbreviations*: HADS-A, Hospital Anxiety and Depression Scale—Anxiety subscale; HADS-D, Hospital Anxiety and Depression Scale—Depression subscale; HADS, Hospital Anxiety and Depression Scale; Mini-MAC, Mini-Mental Adjustment to Cancer scale; PACT, Perceived Ability to Cope with Trauma; BIS, Body Image Scale. ^a^ ES, Effect size (Cohen’s d): d = 0.2 small effect; d = 0.5 medium effect; d = 0.8 large effect [43].

**Table 4:** Pre (T0)–post (T1) comparisons within groups: Paired *t*-tests.

	Intervention Group (n = 21)					Control Group (n = 14)				
Measures (Range)	T0	T1	Paired *t*-Test		ES ^a^	T0	T1	Paired *t*-Test		ES ^a^
	M (SD)	M (SD)	t_(df)_	*p* Value	d	M (SD)	M (SD)	t_(df)_	*p* Value	d
Anxiety: HADS-A (0–21)	7.57 (4.06)	5.29 (2.94)	2.788_(20)_	0.011 *	0.608	9.14 (2.68)	10.57 (3.98)	−1.439_(13)_	0.174	−0.385
Depression: HADS-D (0–21)	4.76 (3.08)	2.95 (2.87)	2.682_(20)_	0.014 *	0.585	6.64 (2.31)	7.14 (3.11)	−0.584_(13)_	0.569	−0.156
Distress: HADS (0–42)	12.33 (5.56)	8.24 (5.33)	3.107_(20)_	0.006 *	0.678	15.79 (4.17)	17.71 (5.24)	−1.422_(13)_	0.179	−0.380
Fighting spirit: Mini-MAC (1–4)	3.06 (0.58)	3.20 (0.65)	−1.474_(20)_	0.156	−0.322	2.95 (0.72)	3.04 (1.00)	−0.330_(13)_	0.747	−0.088
Hopeless/ helplessness: Mini-MAC (1–4)	1.54 (0.52)	1.45 (0.50)	1.789_(20)_	0.089	0.390	1.91 (0.68)	2.96 (0.74)	−5.192_(13)_	0.0001 **	−1.388
Anxious preoccupation: Mini-MAC (1–4)	2.07 (0.67)	1.73 (0.71)	4.198_(20)_	0.0001 **	0.916	2.47 (0.52)	2.64 (0.49)	−1.686_(13)_	0.116	−0.450
Fatalism: Mini-MAC (1–4)	3.04 (0.60)	3.09 (0.50)	−0.384_(20)_	0.705	−0.084	2.92 (0.67)	3.03 (0.79)	−0.591_(13)_	0.565	−0.158
Avoidance: Mini-MAC (1–4)	2.90 (0.82)	2.83 (0.86)	0.487_(20)_	0.631	0.106	2.46 (0.80)	3.32 (0.60)	−3.397_(13)_	0.005 *	−0.908
Forward focus: PACT (1–7)	5.19 (1.12)	5.58 (0.98)	−2.920_(20)_	0.008 *	−0.637	4.50 (1.29)	4.33 (1.34)	0.602_(13)_	0.557	0.161
Trauma focus: PACT (1–7)	4.91 (1.22)	5.17 (0.95)	−1.425_(20)_	0.170	−0.311	4.87 (1.22)	4.36 (1.09)	1.257_(13)_	0.231	0.336
Flexibility: PACT (0–1)	0.86 (0.06)	0.87 (0.06)	−0.818_(20)_	0.423	−0.179	0.82 (0.08)	0.80 (0.08)	0.949_(13)_	0.360	0.254
Body image: BIS (0–30)	14.43 (6.98)	7.95 (3.54)	6.234_(20)_	0.0001 **	1.360	18.64 (5.08)	22.43 (6.11)	−1.717	0.110	−0.459

*Abbreviations*: HADS-A, Hospital Anxiety and Depression Scale—Anxiety subscale; HADS-D, Hospital Anxiety and Depression Scale—Depression subscale; HADS, Hospital Anxiety and Depression Scale; Mini-MAC, Mini-Mental Adjustment to Cancer scale; PACT, Perceived Ability to Cope with Trauma; BIS, Body Image Scale. ^a^ ES, Effect Size (Cohen’s d): d = 0.2 small effect; d = 0.5 medium effect; d = 0.8 large effect [43]. ** *p* < 0.001. * *p* < 0.05.

**Table 5:** Significant moments and important learnings that the participants (n = 21) took home at the end of the Somatic Experiencing^®^ intervention (T1).

Themes	Responses	Meaningful Quotes
**Significant** **moments**	**Responses = 63**	
“Feeling the connection with the body and deep emotional experiences”	51% (n = 32)	*“The first time I entered into a connection with my body and my inner experiences it was as if I knew myself for the first time, or rather, as if I were looking at myself for the first time”.* (ID code: UU121) *“The moment I liked it most was when we experienced grounding. I realized that along with breathing, it is the technique that helps me the most to take care of myself and my needs in everyday life”.* (ID code: AD21I)
“Realizing that it worked”	27% (n = 17)	*“I have always looked forward to each session with joy, I was impatient. It was a meaningful experience. I saw the change in the facial expression of the other women, and so I realized they felt better, we all felt better”.* (ID code: ER13E)
“Reflections in group”	22% (n = 14)	*“I remember with gratitude the first personal reflection in group, it was difficult, but it was also liberating. I felt welcomed, strong, and safe”.* (ID code: RC23A)
**Important** **learnings**	**Responses = 63**	
“Take care of yourself”	57% (n = 36)	*“I realized that my body is a precious ally, I realized that my body does not suck and I should not be afraid. My body is wise and I learned to listen to it to give it, and then give it to myself, what is needed to feel good”.* (ID code: SI22I)
“Live in the ‘here and now”	43% (n = 27)	*“What I learned is ‘take away without necessarily adding’, that is, I learned to live without being always in a hurry and I learned to notice the life around me. […] Now I know the importance of empowering myself to take time to better cope with life’s difficulties”.* (ID code: VR33A).

## Data Availability

The data presented in this study are available on request from the corresponding author, Denise Vagnini, denise.vagnini@unicatt.it.
